# Heart failure with preserved ejection fraction: the impact of right heart failure on risk mortality in hospitalized COVID-19 patients: Cohort study

**DOI:** 10.21542/gcsp.2025.47

**Published:** 2025-10-31

**Authors:** Rocío N. Sánchez Santillán, Arturo Orea-Tejeda, Dulce González-Islas, Celemi Vigil-López, Luz M. Jimenez Gallardo, Nadia Hernandez López, Benigno Valderrabano Salas, Alvaro Montañez Orozco, Tomas Peña Espinosa, Laura P. Arcos Pacheco

**Affiliations:** Heart Failure and Respiratory Distress Clinic, Cardiology Service, Instituto Nacional de Enfermedades Respiratorias “Ismael Cosío Villegas”, Mexico City, Mexico

## Abstract

Introduction: Heart failure is a clinical syndrome characterized by signs and symptoms of structural and/or functional alterations accompanied by impaired expulsion of blood or ventricular filling. COVID-19 contributes to circulatory failure and frequently induces or facilitates pulmonary artery thrombosis. HF can be a risk factor as well as a complication of COVID-19.

Aims: The purpose of this study was to evaluate the association between HFpEF isolate and RHF + HFpEF on mortality according to sex in hospitalized patients caused by COVID-19.

Methods: A prospective cohort study was performed on hospitalized COVID-19 patients from February 2021 to October 2022. Patients ≥18 years with COVID-19 and an echocardiogram during their hospitalization were included. We excluded patients with HIV, shock, and with missing data.

Results: A total of 105 patients were included in this study. The median age was 64 years (50–76), 32% (*n* = 37) of the subjects were women. Patients with “HFpEF + RHF” were found to have an increased risk of death (HR 5.58; 95% CI 1.26–24.6, *p* = 0.023) compared to those with HFpEF alone. A comparison of all patients with HFpEF vs “HFpEF + RHF” according to sex, revealed that women and men with “HFpEF + RHF” had greater mortality than those with HFpEF alone, (*p* = 0.0129, *p* = 0.081), respectively.

Conclusion: Heart failure is an important condition associated with poor prognosis irrespective of the cause of hospitalization. COVID-19 causes damage to the heart and is associated with “RHF and HFpEF”. Furthermore, there were found an increased incidence of “RHF and HFpEF” in women.

## Introduction

Heart failure (HF) is a clinical syndrome characterized by signs and symptoms of structural and/or functional alterations accompanied by impaired expulsion of blood or ventricular filling^1^. On the basis of the left ventricular ejection fraction (LVEF), HF is divided into heart failure with reduced ejection fraction (HFrEF, EF < 40%), heart failure with preserved ejection fraction (HFpEF, EF > 50%) and HF in patients whose ejection fraction (EF) falls between the defining limits of reduced and preserved ejection fraction, or “HF with mild-range EF” (HFmrEF, EF 41–49%). Right heart failure (RHF) may also coexist with structural or functional damage^2^. Recently new diagnostic scoring systems have been proposed for those with preserved EF: the European Society of Cardiology Heart Failure Association PEFF score (HFA-PEFF)^3^ and the American College of Cardiology H2FPEF^4^. This system describes the four stages that focus on the importance of disease development and progression, and each stage is associated with increased mortality^5^.

The main causes of HF are chronic diseases such as myocardial infarction, hypertension, diabetes, obesity, and cardiomyopathy, which gradually have adverse impacts on function. The COVID-19 pandemic highlighted the importance of these conditions, with a prevalence of HF ranging between 4% and 21% in hospitalized patients. Zylla et al.^6^ reported that 3% of patients manifested left ventricular dysfunction after admission, and Chen et al.^7^ found this dysfunction in almost 25% of patients. HF can be a risk factor as well as a complication of COVID-19^8^ and thus increase the risk of mortality.

There are different mechanisms by which COVID-19 can affect cardiac function^9^. COVID-19 contributes to circulatory failure and frequently induces or facilitates pulmonary artery thrombosis^10^. Moreover, hospitalized patients often develop acute respiratory distress syndrome (ARDS)^11^, a condition associated with damage to the right heart. Not infrequently, HFpEF can coexist with RHF and contribute to its progression. However, few studies have explored sex differences related to the coexistence of these two conditions and mortality risk. Therefore, the purpose of this study was to evaluate the association between HFpEF isolate and HFpEF +RHF and mortality according to sex in hospitalized patients caused by COVID-19.

## Material and methods

### Study population and design

A prospective cohort study was performed on hospitalized COVID-19 patients at the Instituto Nacional de Enfermedades Respiratorias “Ismael Cosío Villegas” in Mexico City from February 1st, 2021, to October 31, 2022. All patients ≥18 years old with confirmed COVID-19 via PCR who underwent technically useful echocardiograms during their hospitalization were included. We excluded all patients with HIV, shock at the baseline, and an echocardiography with missing data. Patient demographic characteristics (age, sex), clinical and echocardiographic data were obtained from clinical records collected from a secured web-based electronic database (REDCap).

### Echocardiographic study

All studies were recorded and interpreted offline by a cardiology expert in the field. All echocardiographic parameters were evaluated with a Phillips ”Affiniti 30” color Doppler ultrasound machine.

### Ethics

The study was conducted according to the guidelines of the Declaration of Helsinki. This study was approved by the Institutional Ethics and Research Committee of Biomedical Research in Humans of the Instituto Nacional de Enfermedades Respiratorias “Ismael Cosío Villegas” (approval number E06-20). Due to the nature of the design and according to the General law of health Art. 17, study without risk, no additional informed consent was required.

### Definition of heart failure

At the time of the echocardiographic evaluation, none of the patients had preexisting heart failure. Heart failure with preserved ejection fraction was defined using the diagnostic algorithm from the European Society of Cardiology (ESC). Right heart failure was defined as two of the following: tricuspid annular plane systolic excursion (TAPSE) ≤17 mm and/or right ventricular fractional area change (FAC) < 35% and/or tricuspid regurgitation velocity ≥2.8 mm/seg.

### Endpoint

All patients were followed from the first day in hospital, counting this as the baseline time point, until the last day of hospital stay or death. The primary endpoint was all-cause mortality according to HFpEF and/or HFpEF + RHF.

### Statistical analysis

The statistical analysis was performed using a commercially available STATA version 14 (Stata Corp., College Station, TX, USA). Categorical variables are presented as frequencies and percentages. For the evaluation of normality for continuous variables, the Shapiro−Wilk test was used. Variables with normality are presented as mean and standard deviation, while those with free distribution are presented as median and 25th-75th percentiles.

Baseline characteristics and echocardiographic parameters were divided into groups according to sex. Comparisons among such groups were analyzed with chi-square tests or Fisher’s F tests for categorical variables and independent Student’s *t* tests or Mann−Whitney U tests for continuous variables.

For the primary outcome, we constructed a Cox proportional hazards model and Kaplan–Meier survival graph. The data were divided into two groups, those with HFpEF alone and those with “HFpEF + RHF”, and mortality was compared between them using survival graphs. The Cox proportional hazards model was adjusted for variables with a *p* < 0.20 in the bivariable analysis. Survival plots were evaluated for HFpEF alone vs “HFpEF + RHF”. An exploratory analysis according to sex differences and between patients with HFpEF and those with “HFpEF+RHF” were noted. (Supplementary Material). A log-rank test was performed to compare survival curves between groups. A p <  0.05 was considered to indicate statistical significance.

## Results

A total of 125 patients with COVID-19 were initially included. Three patients were excluded because of septic shock, and 17 patients were excluded because of missing data, resulting in a total of 105 patients in the analysis. 66.7% (*n* = 64) of the participants were male. The median age was 64 (50–76) years, 33.9% with normal weight, 33.0% were overweight and 28.2% were obese, with no differences between sexes. Diffuse interstitial pulmonary disease was more prevalent in women than in men (26.47% vs 6.35%, *p* = 0.010) (Table 1, Supplementary Table 1S).

**Table 1:** Characteristics of the patients at baseline.

Variable	All (*n* = 105)		HFpEF (n=. )		HFpEF + RHF (n= )		p	
Sex								
Male	64 (66.7)		54 (69.2)		10 (55.6)		0.280	
Age (years)	64 (50–76)				
Height (cm)	1.6 (1.53–1.7)				
Weight (kg)	69 (58–83)				
BMI (kg/m^2^)	27.44 ± 6.62				
Underweight <18.5 (kg/m^2^) Normal ≥ 18,5–24.9. Overweight ≥ 25–29.9 Obese ≥ 30	5 (4.9) 35 (33.9) 34 (33.0) 29 (28.2)				
FiO_2_	73.31 ± 13.60				
Comorbidities n (%)								
Diffuse Interstitial pulmonary disease		13 (13.40)					
Cancer		7 (6.80)					
Chronic renal disease		8 (7.77)					
Obstructive Sleep Apnea		7 (6.73)					
Pulmonary Hypertension		3 (2.88)					
Biomass exposure		23 (22.33)					
COPD		9 (8.57)					
Obesity		28 (26.67)					
Systemic Arterial Disease		47 (45.63)					
Diabetes Mellitus		38 (37.62)					
COVID-19 vaccination		65 (65.66)					
Smoker Current Ex smoker		11 (11) 36 (36.4)					
Intrahospital shock		21 (35.59)					
Invasive Mechanical Ventilation		63 (61.76)					
Pneumonia associated with IMV		31 (48.44)					
Acute kidney injury		58 (63.74)					
Hemodialysis		8 (10.26)					
ACE		13 (13.27)					
ARBs		22 (22.45)					
Beta- Blockers		11 (11.34)					
Calcium channel blockers		12 (12.37)					

**Notes.**

ACE Angiotensin converting-enzyme inhibitor ARBs Angiotensin II Receptor Blockers BMI Body Mass Index COPD Chronic Obstructive Pulmonary Disease IMV Invasive Mechanical Ventilation

With respect to the left ventricular ejection fraction, 15 patients (15.6%) had LVEF < 50% and 11 patients (11.5%) had LVEF < 40%, corresponding to HFrEF. 81 patients (64.6%) had LVEF > 50%, and of these 18 patients (18.9%) had RHF. A total of 22 patients (22.4%) were found to have RHF (Tables 2 and 3). Additionally, we explore differences between HFpEF vs HFpEF + RHF and survivors to evaluate any cofounder variable and independence of data.

**Table 2:** Distribution of Heart Failure Subtypes.

**Heart Failure Subtype**	**n (%)**
Total	96 (100)
HFmrEF/HFrEF (LVEF < 50%)	15 (15.6)
HFpEF (LVEF ≥ 50%)	62 (64.6)
HFpEF with RHF	18 (18.9)
RHF	22 (22.9)

**Table 3:** Echocardiographic parameters according to MO.

Variable	All (n=)	Survivors	Non-Survivors	p
HF mild and reduce EF	15 (15.8)	11 (14.1)	4 (23.5)	0.417
HFpEF (LVEF > 50%)	62 (65.2)	53 (68)	9 (53)	
HFpEF + RHF	18 (19)	14 (17.9)	4 (23.5)	

When echocardiographic parameters were evaluated for sex, there were expected differences in ventricular diameters, nonetheless, these differences remained within the normal range (Table S2). Evidence of increased filling pressure was evaluated according to the HFA-PEFF diagnostic algorithm from the ESC^12^. There were no statistical differences between the sex groups. (Table S3)

A Cox proportional model was constructed to evaluate mortality in patients with HFpEF alone and in patients with HFpEF + RHF. Patients with ”HFpEF + RHF” were found to have an increased risk of death (HR 5.58; 95% CI 1.26–24.6, *p* = 0.023) compared to those with HFpEF alone. The model 1 was adjusted for sex, partial thromboplastin time, and D -dimer concentration according to variables with *p*<0.20 in the bivariate analysis. Model 2 was adjusted for age, partial thromboplastin time and D Dimer with a HR of 5.15 (95% CI 1.17–22.6, *p* = 0.030) (Table 4).

**Table 4:** Hazard ratio for primary outcome.

Variable	Crude model		Adjusted model 1		Adjusted model 2	
	HR (IC 95%)	p	HR (CI 95%)	p	HR (CI 95%)	p
Age (years)	1.04 (0.99–1.09)	0.067	–	–	1.04 (1.17-22.6)	0.030
Sex	a0.39 (0.13–1.18)	0.096	0.37 (0.10-1.32)	0.128	–	–
Cancer	4.73 (1.23–18.16)	0.023	–	–	–	–
Diffuse Interstitial pulmonary disease	4.89 (1.15–20.77)	0.031	–	–	–	–
IMV	0.31 (0.10–0.99)	0.050	–	–	–	–
RHF	2.24 (0.66–7.53)	0.190	–	–	–	–
HFpEF+RHF HFpEF	2.36 (0.71–7.74) 0.42 (0.12–1.39)	0.157	5.58 (1.26–24.6)	0.023	5.15 (1.17–22.6) 0.19 (0.04–0.85)	0.030
PTT	0.95 (0.89–1.00)	0.095	0.92 (0.86–0.99)	0.012	0.91 (0.84–0.99)	0.030
D Dimer	1.04 (0.99–1.10)	0.085	1.08 (1.01–1.15)	0.041	1.05 (0.98–1.12)	0.112
ARB‘s	0.22 (0.02-1.74)	0.152	–	–	–	–

**Notes.**

ARB’s Angiotensin II Receptor Blockers HFpEF Heart Failure with Preserved Ejection Fraction IMV Invasive Mechanical Ventilation RHF Right Heart Failure PTT Partial Thromboplastin Time

LLR= −27.073 *n* = 59 ; LLR= −26.997.

**Figure 1.**
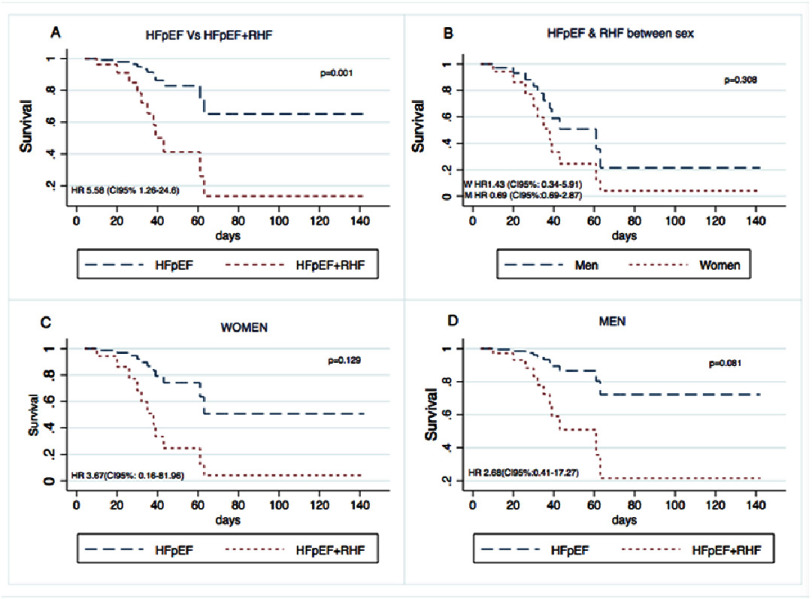
Kaplan Meier Survival Curves from patients with HFpEF and “HFpEF + RHF”. (1A) Comparison between all the sample, HFpEF vs “HFpEF + RHF”. (1B) Comparison between men and women diagnosed with “HFpEF + RHF” (*n* = 22). (1C) Comparison between women diagnosed with HFpEF vs “HFpEF + RHF”. (1D) Comparison between men diagnosed with HFpEF vs “HFpEF + RHF”. HFpEF, Heart failure with Preserved Ejection Fraction; RHF, Right Heart Failure.

Patients with “HFpEF + RHF” had greater mortality than did those with HFpEF alone (*p*<0.01) (Figure 1A). Among those with “HFpEF and RHF”, women had a greater mortality rate than men did (*p* = 0.308) (Figure 1B). A comparison of all patients with HFpEF vs “HFpEF + RHF” according to sex, revealed that women with “HFpEF + RHF” had greater mortality than women with HFpEF alone (*p* = 0.129) (Figure 1C). Similar data were found between men with greater mortality in those with “HFpEF + RHF” (*p* = 0.081) (Figure 1D), although the last three comparisons did not reach statistical significance due to the sample size.

## Discussion

Although the morbi-mortality of HFrEF has been studied extensively in the literature, the impact of HFpEF on survival remains controversial, and that of RHF is even more limited. The purpose of this study was to evaluate the impact of HFpEF vs “HFpEF + RHF” on mortality, and to explore the role of sex. Among a total of 105 hospitalized COVID-19 patients, 11.5% had an LVEF< 40%, 4.1% had a LVEF between 40 and 49%, and 84.4% had a LVEF > 50%. Regarding right heart function, 17.8%^18^ had a TAPSE < 17 mm, and 12.4%^13^ had a FAC < 35%. A total of 24.4% had RHF. The mortality rate was greater in patients with concomitant HFpEF and RHF than in those with HFpEF alone.

It is important to note the high prevalence of HFpEF associated with high mortality, as HF with a reduced ejection fraction was considered to confer a worse prognosis. At least in women hospitalized with COVID-19, HFpFE had the highest risk of mortality in the first sixty days, especially when it was associated with RHF, as occurred in almost 19% of patients. The comorbidities were similar between the sexes, although in the case of women, one outstanding difference was the high prevalence of diffuse interstitial pulmonary disease (almost 5 times more frequent than in men) which could explain why the highest mortality occurred in patients with greater prior lung damage.

Preexisting HF prevalence data worldwide range from 1 to 21%, and new diagnoses of LV dysfunction range between 3% and 25%^6,8,13,14^. The overall mortality rate ranged between 20% and 40% in hospitalized COVID-19 patients with any type of HF; in our study, the mortality rate was consistent with that in previous reports. It is difficult to compare data on preexisting HF with recently or previously diagnosed RHF, especially among patients with COVID-19 infection with or without hospitalization.

As previously noted, COVID-19 may induce severe acute cardiac injury accompanied by alterations in cardiac function, resulting in HF and RHF. Rath et al. discovered that patients with COVID-19 infection and impaired left ventricular and right ventricular function as well as tricuspid regurgitation were significantly associated with a worse prognosis when infected and hospitalized for COVID-19. Although our research does not have the same objective, right heart damage to the heart can be demonstrated to result in a worse prognosis and increased mortality in patients of both sexes.

Several mechanisms may contribute to cardiac injury and heart failure in this population. First, preexisting cardiovascular risk factors—including diabetes mellitus, hypertension, and obesity—are prevalent in the study population and classify these patients as Stage A (at risk for heart failure) according to American Heart Association/American College of Cardiology (AHA/ACC) guidelines. Second, direct SARS-CoV-2-mediated injury to endothelial cells triggers activation of inflammatory mediators, increased thrombotic activity, direct myocardial injury, and renin-angiotensin-aldosterone system activation. These pathophysiological processes collectively lead to myocardial depression, volume overload, and elevated afterload, ultimately resulting in right ventricular failure.

Several studies have documented right ventricular injury in patients with COVID-19, with elevated cardiac troponin levels traditionally associated with left ventricular ischemia or infarction. However, elevated serum troponin levels have been observed in 25.7% of heart failure patients with preserved ejection fraction, attributed to myocyte membrane disruption and release of contractile proteins into the circulation^16^.

However, evidence in patients with COVID-19 suggests that the primary mechanism of troponin elevation is right ventricular injury. Our findings demonstrated that patients with right heart failure had a fivefold increased risk of mortality, indicating that COVID-19 can cause myocardial damage. Furthermore, 72.2% of patients with right heart failure had elevated troponin levels >14.8 pg/mL. When comparing median troponin levels across heart failure subtypes, significant differences were observed: HFpEF versus RHF showed median values of 8.0 (IQR 1.8–58.7) pg/mL versus 73.3 (IQR 14.8–394.7) pg/mL (*p* = 0.001). Across all three groups—HFrEF, HFpEF, and HFpEF with RHF—median troponin levels were 31.3 (IQR 14.2–199.4), 8.0 (IQR 1.8–58.7), and 48.1 (IQR 14.8–394.7) pg/mL, respectively, further supporting the presence of right ventricular injury (Table 2S)^17,18^.

Right ventricular abnormalities, as an independent parameter, indicate poor prognosis in patients with COVID-19. Li et al. reported that tricuspid annular plane systolic excursion, right ventricular fractional area change, and right ventricular longitudinal strain were predictors of mortality^19^. Consistent with our findings, right ventricular dysfunction is frequently associated with more severe clinical manifestations and complications, including renal dysfunction and pulmonary hypertension, resulting in worse outcomes^20–22^.

Another cardiovascular biomarker is B-type natriuretic peptide (BNP) or N-terminal pro-B-type natriuretic peptide (NT-proBNP), which are secreted by atrial and ventricular myocytes in response to increased volume or pressure loading. These biomarkers represent an important diagnostic variable in the Heart Failure Association heart failure with preserved ejection fraction (HFA-PEFF) algorithm. In our cohort, 72% of subjects had BNP levels ≥35 pg/mL, meeting the diagnostic criteria for the HFA-PEFF algorithm.

Our data demonstrated in-hospital mortality rates of 15.6% for heart failure with mildly reduced and reduced ejection fraction, 64.7% for HFpEF, 22.9% for right heart failure, and 18.9% for combined HFpEF with right heart failure.

Our overall mortality rate was lower than that reported by Goyal et al.^15^, who documented in-hospital mortality rates of 35% for HFrEF, 27.1% for HFmrEF, 30.4% for HFpEF, and 31.7% for an unspecified subtype; however, they did not evaluate right heart failure separately. For the association between in-hospital mortality and heart failure subtype, they constructed a fully adjusted regression model yielding the following relative risks: HFrEF, RR 1.40 (95% CI 1.10–1.79); HFmrEF, RR 1.06 (95% CI 0.65–1.73); HFpEF, RR 1.08 (95% CI 0.84–1.33); and unspecified subtype, RR 1.16 (95% CI 1.02–1.32). In contrast, our patients with combined HFpEF and right heart failure demonstrated significantly greater risk of in-hospital mortality (HR 5.58, 95% CI 1.26–24.6), which may be attributed to differences in patient populations and effect measures used.

This may be explained by the preexisting lung damage in 26% of the women, which contributed to increased PASP and RHF development as well as to the damage caused by SARS-CoV-2.

When comparing mortality between patients with HFpEF versus combined HFpEF with right heart failure, we observed a statistically significant difference in in-hospital mortality among patients with COVID-19 (Figure 1). Similar findings were reported by Panagides et al. in France^23^. However, they compared the hazard ratio between patients with HFpEF and those with HFrEF, demonstrating an HR of 1.61 (95% CI 1.13–2.27; *p* = 0.01) for HFpEF^23^.

We hypothesize that the higher mortality rate observed in patients with combined HFpEF and right heart failure may be attributed to two primary mechanisms. First, direct SARS-CoV-2-mediated injury to the right ventricle occurs in the absence of established treatment guidelines for right heart failure. Second, physiological overload of the right ventricle results from elevated postcapillary pressure secondary to left ventricular failure, indirectly compromising right ventricular function.

**Figure 2.**
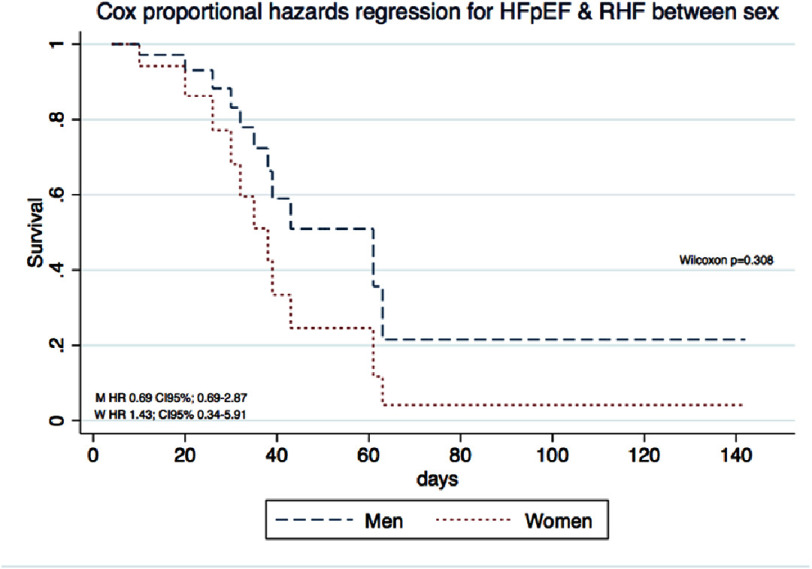
Cox proportional hazards regression for HFpEF & RHF between sexes.

**Figure 3.**
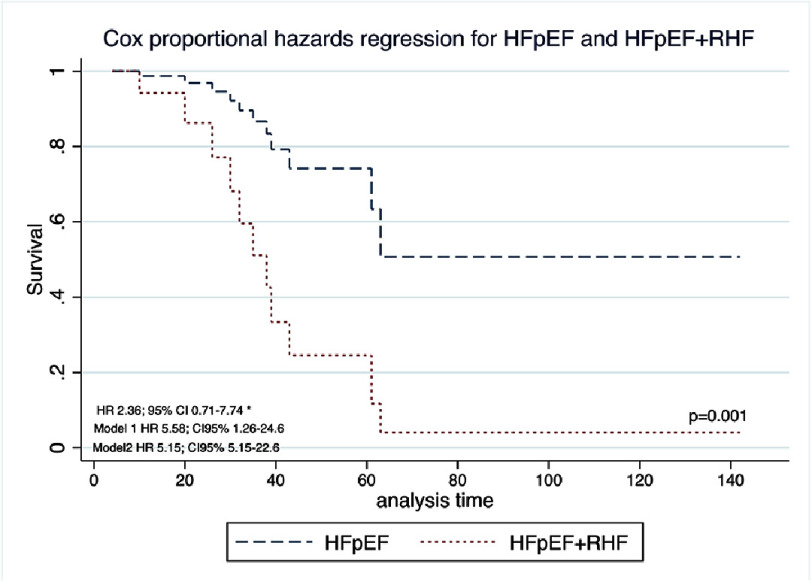
Cox proportional hazards regression for HFpEF and HFpEF+RHD amongst females.

Survival curves demonstrated consistent differences between patients with HFpEF and those with combined HFpEF and right heart failure. When stratified by sex, women exhibited earlier mortality than men (Figure 2), and among women, those with combined HFpEF and right heart failure had worse survival compared to those with HFpEF alone (Figure 3). However, these differences did not reach statistical significance due to limited sample size. While HFpEF is known to be more prevalent in women and HFrEF more prevalent in men, we observed no sex-based difference in HFpEF prevalence. This finding may be explained by COVID-19-induced myocardial injury superseding traditional sex-related differences in heart failure phenotype^24^.

In exploratory sex-stratified analyses, women demonstrated shorter survival compared to men. COVID-19 affects cardiac function through both direct and indirect mechanisms. Inflammation and immune system dysregulation may contribute to heart failure development. Additionally, preexisting comorbidities—including hypertension, diabetes mellitus, atrial fibrillation, obesity, and coronary artery disease—are established risk factors associated with HFpEF development and adverse prognosis. Notably, hypertension and diabetes mellitus are more prevalent in women than in men (diabetes prevalence: 5.3% vs 2.4%, *p* < 0.01)^24,25^. This sex-based disparity in comorbidity burden may explain the increased mortality risk observed in women with HFpEF and right heart failure compared to men.

HF remains a significant cause of in-hospital mortality. While HFpEF was historically underrecognized, it has gained increasing attention in recent years. The incidence of HFpEF continues to rise, as COVID-19 can cause myocardial injury, contributing to HFpEF development along with right ventricular dysfunction and dilation. The presence of these combined conditions substantially increases mortality risk.

Additionally, demographic shifts have resulted in a greater burden of chronic disease, contributing to increased HFpEF incidence. With the emergence of novel pathogens such as SARS-CoV-2, which cause both direct and indirect myocardial injury, we anticipate a rising incidence of concurrent HFpEF and right heart failure.

## Limitations

The primary limitation of this study is the small sample size. Echocardiographic data were limited for patients with COVID-19, as direct patient contact was restricted early in the pandemic to minimize infection risk to healthcare personnel.

For HFpEF diagnosis, the ESC guidelines identify BNP as an important biomarker that should be routinely measured. However, the ESC scoring algorithm can be applied even when not all parameters are available. We applied this approach to our cohort; despite incomplete BNP data for all patients, our findings demonstrate a high prevalence of HFpEF and right heart failure. This indicates that the ESC diagnostic algorithm can be feasibly implemented in resource-limited settings.

## Conclusion

Heart failure is an important condition that is associated with poor prognosis irrespective of the cause of hospitalization. COVID-19 causes damage to the heart and is associated with RHF and HFpEF. These conditions are more common now and are associated with an increased mortality risk in hospitalized patients with COVID-19. Furthermore, there are sex differences in heart and lung diseases, which can lead to an increased incidence of RHF and HFpEF in women in this study, as well as a worse prognosis.

## Authors’ contributions

**Conceptualization**, RNSS and AOT; **Methodology**, DGI, RNSS; **Validation**, RNSS, AOT, DGI, CVL, LMJG, NHL, BVS, AMO, TPE, LPAP; **Formal Analysis**, RNSS; **Investigation**, RNSS., DGI, CVL, TPE, LPAP; **Resources**, RNSS, DGI, AOT, BVS, AMO and ARP; **Resources**; **Data** **Curation**, RNSS, and DGI; **Writing** –**Original** **Draft** **Preparation**, RNSS, AOT; **Writing –Review & Editing**, RNSS, AOT, DGI, LMJG; **Visualization**, RNSS, AOT, DGI, LPAP; **Supervision**, RNSS, AOT, DGI ; **Project Administration**. RNSS, AOT, DGI; **Supervision**, RNSS, AOT, DGI.

## Funding

This research did not receive any specific grant from funding agencies in the public, commercial, or not-for-profit sectors.

## Conflict of interest

None declared

## Data availability statement

The datasets analyzed during the current study are available upon reasonable request. Contact rnsanchezs@gmail.com

## Abbreviations

ACE, angiotensin-converting enzyme; ARBs, angiotensin II receptor blockers; ARDS, acute respiratory distress syndrome; BMI, body mass index; BSA, body surface area; COPD, chronic obstructive pulmonary disease; DTSV, right ventricular outflow tract diameter; EF, ejection fraction; ESC, European Society of Cardiology; FAC, right ventricular fractional area change; HF, heart failure; HFA-PEFF, European Society of Cardiology Heart Failure Association PEFF score; HFrEF, heart failure with reduced ejection fraction (EF < 40%); HFpEF, heart failure with preserved ejection fraction (EF ≥50%); HFmEF, heart failure with mid-range ejection fraction (EF 41–49%); IVC, inferior vena cava; IVS, interventricular septum; LA, left atrial; LV, left ventricle; LVEF, left ventricular ejection fraction; LVOT, left ventricular outflow tract; LV TDV, left ventricular tele-diastolic volume; LV TSV, left ventricular tele-systolic volume; MRA, mineralocorticoid receptor antagonist; PASP, pulmonary arterial systolic pressure; PVAT, pulmonary velocity acceleration time; RA, right atrial; REDCap, Research Electronic Data Capture; RHF, right heart failure; TAPSE, tricuspid annular plane systolic excursion; TR-Vmax, tricuspid regurgitant gradient velocity; VTI, velocity time integral.
